# Supplementary material for: Comparison of life history parameters of two different genetic clusters of Bemisia tabaci MED (Hemiptera: Aleyrodidae) through single and cross mating
Source: PLoS One. 2021 Mar 26;16(3):e0248819. doi: 10.1371/journal.pone.0248819 (PMC7997046; doi:10.1371/journal.pone.0248819)
Supplement: S1 Table — (DOCX) [file pone.0248819.s001.docx]

S1 Table. Genetic diversity of the *B. tabaci* MED treatments

| **Host plant** | **Treatment** | **N** | ***N*_A_** | ***H*_E_** | ***H*_O_** | ***F*_IS_** |
| --- | --- | --- | --- | --- | --- | --- |
| Cucumber | C1 | 20 | 2.875 | 0.437 | 0.100 | 0.771 |
|  | C2 | 20 | 2.875 | 0.400 | 0.081 | 0.797 |
|  | C1fC2m | 20 | 5.625 | 0.710 | 0.231 | 0.674 |
|  | C2fC1m | 20 | 5.500 | 0.672 | 0.288 | 0.572 |
| Tobacco | C1 | 20 | 4.625 | 0.550 | 0.113 | 0.796 |
|  | C2 | 20 | 4.500 | 0.497 | 0.075 | 0.849 |
|  | C1fC2m | 20 | 5.750 | 0.665 | 0.288 | 0.568 |
|  | C2fC1m | 20 | 6.125 | 0.685 | 0.263 | 0.617 |

N, number of individuals sampled; *N*_A_, Mean number of alleles per population; *H*_E_, Mean expected heterozygosity; *H*_O_, Mean observed heterozygosity; and *F*_IS_, Mean fixation index inbreeding coefficient. Significance *F*_IS_ value is obtained after 1,000 permutation tests.
