# Supplementary material for: Comparison of life history parameters of two different genetic clusters of Bemisia tabaci MED (Hemiptera: Aleyrodidae) through single and cross mating
Source: PLoS One. 2021 Mar 26;16(3):e0248819. doi: 10.1371/journal.pone.0248819 (PMC7997046; doi:10.1371/journal.pone.0248819)
Supplement: S2 Table — (DOCX) [file pone.0248819.s002.docx]

S2 Table. Results of two-way ANOVA for testing effects of cluster and host plant on biological characteristics, body weight, and body length of *B. tabaci*

| **Parameter** | **Source** | **df** | **MS** | ***F*** | ***p*** |
| --- | --- | --- | --- | --- | --- |
| Total fecundity | Cluster | 3 | 150130.049 | 538.85 | < 0.0001 |
|  | Host | 1 | 803337.004 | 288.35 | < 0.0001 |
|  | Cluster × Host | 3 | 11381.782 | 40.85 | < 0.0001 |
|  | Error | 232 | 278.610 |  |  |
| Daily fecundity | Cluster | 3 | 94.110 | 227.15 | < 0.0001 |
|  | Host | 1 | 78.296 | 188.98 | < 0.0001 |
|  | Cluster × Host | 3 | 9.848 | 23.77 | < 0.0001 |
|  | Error | 232 | 0.414 |  |  |
| Female  longevity | Cluster | 3 | 1887.989 | 171.18 | < 0.0001 |
|  | Host | 1 | 0.067 | 0.01 | 0.9381 |
|  | Cluster × Host | 3 | 10.922 | 0.99 | 0.3981 |
|  | Error | 232 | 11.029 |  |  |
| Oviposition  period | Cluster | 3 | 1327.304 | 135.45 | < 0.0001 |
|  | Host | 1 | 5.704 | 0.58 | 0.4463 |
|  | Cluster × Host | 3 | 13.126 | 1.34 | 0.2623 |
|  | Error | 232 | 9.800 |  |  |
| Post-oviposition  period | Cluster | 3 | 70.315 | 28.40 | < 0.0001 |
|  | Host | 1 | 7.004 | 2.83 | 0.0940 |
|  | Cluster × Host | 3 | 12.515 | 5.05 | 0.0021 |
|  | Error | 232 | 2.476 |  |  |
| Survival rate of  immature stage | Cluster | 3 | 64.121 | 4.56 | 0.0040 |
|  | Host | 1 | 4.637 | 0.33 | 0.5663 |
|  | Cluster × Host | 3 | 234.644 | 16.70 | < 0.0001 |
|  | Error | 232 | 14.053 |  |  |
| Sex ratio | Cluster | 3 | 5234.002 | 1028.43 | < 0.0001 |
|  | Host | 1 | 282.528 | 55.51 | < 0.0001 |
|  | Cluster × Host | 3 | 287.260 | 56.44 | < 0.0001 |
|  | Error | 232 | 5.089 |  |  |
| Developmental period  (Female + Male) | Cluster | 3 | 577.018 | 744.40 | < 0.0001 |
|  | Host | 1 | 4.210 | 5.43 | 0.0203 |
|  | Cluster × Host | 3 | 0.440 | 0.57 | 0.6368 |
|  | Error | 362 | 0.775 |  |  |
| Developmental period  (Female) | Cluster | 3 | 188.633 | 1080.82 | < 0.0001 |
|  | Host | 1 | 2.311 | 13.24 | 0.0004 |
|  | Cluster × Host | 3 | 1.242 | 7.11 | 0.0001 |
|  | Error | 191 | 0.175 |  |  |
| Developmental period  (Male) | Cluster | 3 | 236.876 | 549.22 | < 0.0001 |
|  | Host | 1 | 1.311 | 3.04 | 0.0832 |
|  | Cluster × Host | 3 | 0.280 | 0.65 | 0.5852 |
|  | Error | 163 | 0.431 |  |  |
| Body weight  (Female) | Cluster | 3 | 0.020 | 128.13 | < 0.0001 |
|  | Host | 1 | 0.001 | 3.16 | 0.0795 |
|  | Cluster × Host | 3 | 0.000 | 1.46 | 0.2326 |
|  | Error | 72 | 0.000 |  |  |
| Body weight  (Male) | Cluster | 3 | 0.031 | 343.75 | < 0.0001 |
|  | Host | 1 | 0.000 | 4.18 | 0.0444 |
|  | Cluster × Host | 3 | 0.000 | 0.65 | 0.5862 |
|  | Error | 72 | 0.000 |  |  |
| Body length  (Female) | Cluster | 3 | 0.759 | 654.17 | < 0.0001 |
|  | Host | 1 | 0.001 | 0.87 | 0.3504 |
|  | Cluster × Host | 3 | 0.000 | 0.18 | 0.9118 |
|  | Error | 792 | 0.001 |  |  |
| Body length  (Male) | Cluster | 3 | 1.088 | 485.70 | < 0.0001 |
|  | Host | 1 | 0.006 | 2.65 | 0.1038 |
|  | Cluster × Host | 3 | 0.002 | 0.88 | 0.4525 |
|  | Error | 792 | 0.002 |  |  |
