# Supplementary material for: Comparison of life history parameters of two different genetic clusters of Bemisia tabaci MED (Hemiptera: Aleyrodidae) through single and cross mating
Source: PLoS One. 2021 Mar 26;16(3):e0248819. doi: 10.1371/journal.pone.0248819 (PMC7997046; doi:10.1371/journal.pone.0248819)
Supplement: S3 Table — (DOCX) [file pone.0248819.s003.docx]

S3 Table. Tomato yellow leaf curl virus detected of *B. tabaci* MED populations in Korea from 2016 to 2018

| **No.** | **Population** | **2016** | **2017** | **2018** |
| --- | --- | --- | --- | --- |
| 1 | CC | 0.00 | 0.00 |  |
| 2 | PT | 0.00 | 0.26 | 0.01 |
| 3 | SJ | 0.00 | 0.01 | 3.46 |
| 4 | CY | 28.10 | 1.80 |  |
| 5 | BY | 0.00 |  |  |
| 6 | IS | 0.06 | 0.00 |  |
| 7 | JE | 9.45 | 0.00 |  |
| 8 | GJ | 0.10 | 0.00 |  |
| 9 | BS | 243.90 | 24.10 | 171.25 |
| 10 | SC | 18.30 | 1.60 | 4.92 |
| 11 | JIN | 33.10 | 0.00 |  |
| 12 | CW | 0.00 |  |  |
| 13 | GH | 3.34 |  |  |
| 14 | MY | 0.00 | 0.00 |  |
| 15 | AD | 10.48 |  |  |
| 16 | BUS | 0.80 | 1.70 |  |
| 17 | JJ | 1.33 | 0.00 | 40.79 |
