# Supplementary material for: Comparison of life history parameters of two different genetic clusters of Bemisia tabaci MED (Hemiptera: Aleyrodidae) through single and cross mating
Source: PLoS One. 2021 Mar 26;16(3):e0248819. doi: 10.1371/journal.pone.0248819 (PMC7997046; doi:10.1371/journal.pone.0248819)
Supplement: S4 Table — (DOCX) [file pone.0248819.s004.docx]

S4 Table. *Wolbachia* detected of *B. tabaci* MED populations in Korea from 2016 to 2018

| **No.** | **Population** | **2016** | **2017** | **2018** |
| --- | --- | --- | --- | --- |
| 1 | CC | - | - |  |
| 2 | PT | - | - | - |
| 3 | SJ | - | - | - |
| 4 | CY | - | - |  |
| 5 | BY | + |  |  |
| 6 | IS | - | - |  |
| 7 | JE | - | - | - |
| 8 | GJ | - | - |  |
| 9 | BS | - | - |  |
| 10 | SC | - | - | - |
| 11 | JIN | - | - |  |
| 12 | CW | - | - |  |
| 13 | GH | - |  |  |
| 14 | MY | - | - |  |
| 15 | AD | - |  |  |
| 16 | BUS | - | - |  |
| 17 | JJ | - | - | - |
