# Supplementary material for: Comparison of life history parameters of two different genetic clusters of Bemisia tabaci MED (Hemiptera: Aleyrodidae) through single and cross mating
Source: PLoS One. 2021 Mar 26;16(3):e0248819. doi: 10.1371/journal.pone.0248819 (PMC7997046; doi:10.1371/journal.pone.0248819)
Supplement: Attachment — Submitted filename: PLOSONE 30 12 2020.docx [file pone.0248819.s005.docx]

The provided abstract does not adequately represent the manuscript

The introduction is poorly written
