# Supplementary material for: Comparison of life history parameters of two different genetic clusters of Bemisia tabaci MED (Hemiptera: Aleyrodidae) through single and cross mating
Source: PLoS One. 2021 Mar 26;16(3):e0248819. doi: 10.1371/journal.pone.0248819 (PMC7997046; doi:10.1371/journal.pone.0248819)
Supplement: Attachment — Submitted filename: Manuscript PONE-D-20-28992 - Notes from reviewer.doc [file pone.0248819.s006.doc]

**Comparison of life history characteristics of two different genetic clusters of Bemisia tabaci MED (Hemiptera: Aleyrodidae)**

by: Yujeong Park, Young-Gyun Park and Joon-Ho Lee

**REVIEWER’S NOTES**

The manuscript reports an interesting research, which provides light on genetic dynamics in two populations of a species of the *Bemisia tabaci* group having a relevant applied and economic interest in vast geographical areas. The studies have been adequately structured and planned in the methodology, producing results consistent with the experiment and conclusions adequate to the results obtained. Therefore, the paper is worth of being published on PlosOne, after only very minor changes. In particular, in addition to detecting a few small weakness regarding literature references in some parts of the paper, I am pointing out an aspect that makes me quite dubious and to which I would like to draw the Author's attention (see “main note” below).

**MAIN NOTE**

Stating that I am not a geneticist, I admit I do not know whether the terminology used in the paper is adequate. However, as a "non-geneticist entomologist", I have the feeling that the term "convergence" is not the most convenient to indicate the phenomenon of prevalence of Cluster C1 over Cluster C2. With reference to two distinct elements, A and B, the term convergence indicates, in my opinion, a reciprocal dynamism of the two elements, with each one going in the direction of the other; while, in the specific case presented in this paper, one element prevails over the other. In this case, can the term "convergence" be used? Or would it not be better to use the term "displacement" to indicate the dominance of Cluster C1 over Cluster C2 and the progressive reduction of the latter?

Please, be aware, this does not want to be a criticism but simply a doubt and “food for thought”.

Anyway, if my doubts on the term “convergence” are accepted by the Authors, they should modify accordingly the term in many lines of the paper, such as: lines 19, 32, 44, 274, 281, 299.

**SPECIFIC NOTES**

Line 39: Add “,”after “Aleyrodidae)”.

Line 40: literature cited [1-3] is almost scarce and of limited importance for the phrase quoted. Above all, paper no. 1 (Chen et al., 2004) and no. 3 (Tsagkarakou et al., 2012) refer to extremely detailed and particular aspects (for both host plants and diseases caused, as well as for geographic distribution cited); therefore, they are not well suited to being cited as example of references to the high harmfulness of *B. tabaci* at a broad geographical level and on a wide range of crops (these two citations could be deleted, eventually). Paper no. 2 (Navas Castillo et al., 2011) is better suited to this aim and should be maintained, definitively. But many other papers could be added, dealing with harmfulness of *B. tabaci* worldwide, on wide geographical areas and cropping systems. Examples:

- Lowe S, Browne M, Boudjelas S, De Poorter M, 2000. *100 of the World’s Worst Invasive Alien Species – A Selection from the Global Invasive Species Database*. The Invasive Species Specialist Group (ISSG), Species Survival Commission (SSC), Hollands Printing Ltd: 12 pp.
- Martin JH, Mifsud D, Rapisarda C, 2000. The whiteflies (Hemiptera: Aleyrodidae) of Europe and the Mediterranean Basin. *Bulletin of Entomological Research*, 90: 407-448.
- Legg JP, Shirima R, Tajebe LS, Guastella D, Boniface S, Jeremiah S, Nsami E, Chikoti P, Rapisarda C, 2014. Biology and management of *Bemisia* whitefly vectors of cassava virus pandemics in Africa. *Pest Management Science*, 70 (10): 1446-1453.
- Polston JE, De Barro P, Boykin LM, 2014. Transmission specificities of plant viruses with the newly identified species of the *Bemisia tabaci* species complex. *Pest Management Science*, 70: 1547–1552.

Line 271: literature cited [12, 13, 34-38] is not satisfactory and updated; in general, it is not giving the right and complete idea of the huge scientific production on biology of different putative species of *B. tabaci* on different host plants and at varying temperatures. I strongly suggest to include also the following references (or at least the most recent ones, citing most of the previous papers):

- Nava-Camberos U, Riley DG, Harris MK, 2001. Temperature and host plant effects on development, survival, and fecundity of *Bemisia argentifolii* (Homoptera: Aleyrodidae). *Environmental Entomology*, 30 (1): 55-63.
- Qui B, Shun-Xiang R, Mandour NS, Li L, 2003. Effect of temperature on the development and reproduction of *Bemisia tabaci* B biotype (Homoptera: Aleyrodidae). *Insect Science*, 10 (1): 43-49.
- Bayhan E, Ulusoy MR, Brown JK, 2006. Effects of different cucurbit species and temperature on selected life history traits of the ‘B’biotype of *Bemisia tabaci*. *Phytoparasitica*, 34 (3): 235-242.
- Yang TC, Chi H, 2006. Life tables and development of *Bemisia argentifolii* (Homoptera: Aleyrodidae) at different temperatures. *Journal economic Entomology*, 99 (3): 691-698.
- Bonato O, Lurette A, Vidal C, Fargues J, 2007. Modelling temperature-dependent bionomics of *Bemisia tabaci* (Q-biotype). *Physiological Entomology*, 32 (1): 50-55.
- Xie M, Wan FH, Chen YH, Wu G, 2011. Effects of temperature on the growth and reproduction characteristics of *Bemisia tabaci* B-biotype and *Trialeurodes vaporariorum*. *Journal applied Entomology*, 135 (4): 252-257.
- Guo JY, Cong L, Wan FH, 2013. Multiple generation effects of high temperature on the development and fecundity of *Bemisia tabaci* (Gennadius) (Hemiptera: Aleyrodidae) biotype B. *Insect Science*, 20 (4): 541-549.
- Han EJ, Choi BR, Lee JH, 2013. Temperature-dependent development models of *Bemisia tabaci* (Gennadius) (Hemiptera: Aleyrodidae) Q biotype on three host plants. *Journal Asia Pacific Entomology*, 16 (1): 5-10.
- Aregbesola OZ, Legg JP, Sigsgaard L, Lund OS, Rapisarda C, 2019. Potential impact of climate change on whiteflies and implications for the spread of vectored viruses. *Journal of Pest Science*, 92: 381-392.
- Aregbesola OZ, Legg JP, Lund OS, Sigsgaard L, Sporleder M, Carhuapoma P, Rapisarda C, 2020. Life history and temperature‑dependence of cassava‑colonising populations of *Bemisia tabaci*. *Journal of Pest Science*, 93: 1125-1241.
