# Supplementary material for: Comparison of life history parameters of two different genetic clusters of Bemisia tabaci MED (Hemiptera: Aleyrodidae) through single and cross mating
Source: PLoS One. 2021 Mar 26;16(3):e0248819. doi: 10.1371/journal.pone.0248819 (PMC7997046; doi:10.1371/journal.pone.0248819)
Supplement: Attachment — Submitted filename: Response letter.docx [file pone.0248819.s007.docx]

**Dear Editor and reviewer.**

We are grateful for the reviewer’s valuable comments to improve our manuscript. We improved our manuscript significantly by incorporating the reviewer’s comments and suggestions. We have colored revised parts in red in the manuscript. Followings are our point-by-point responses to the comments:

**Reviewer #1:**

Thank you for the specific comments on our study.

L1 (Title): please replace "life history characteristics" with "life history parameters"

Our response: Corrected.

L2: for a sufficiently informative title, please add "through single and cross mating" after "(Hemiptera: Aleyrodidae)"

Our response: Corrected.

L16-17: change "is one of serious insect pests with economic importance worldwide" to "is an economically important insect pest worldwide"

Our response: Corrected.

L18-19: change "on greenhouse tomatoes" to "occurring in greenhouse tomatoes"

Our response: Corrected.

L20: one-year period

Our response: Corrected.

L21: replace (here and wherever possible throughout the manuscript) "characteristics" with "parameters"

Our response: Corrected.

L22: please delete "of *B. tabaci* MED" as you already stated this earlier

Our response: Corrected.

L41: please specify which kind of "regions" ? (cultivated with which species of host crops); or you simply may change to "agricultural-producing regions"

Our response: We added a new sentence additional explanation after this sentence (Lines 41-44).

L41: add a comma before "whereas"

Our response: Corrected.

L53: replace "Especially" with "More specifically"

Our response: Corrected.

L55: change "to find evidence" with "to provide more evidence"

Our response: Corrected.

L79: replace "These two plants belong to the" with "These two species are among the"

Our response: Corrected.

L82: *Bemisia tabaci* colonies were ........ (always write species in full when starting a new sentence)

Our response: Corrected.

L92: (< 12-h-old)

Our response: Corrected.

L98: before the experiments were initiated.

Our response: Corrected.

Page 13 - TABLE 1 (third column): Sing cluster ?? did you mean "Single cluster" ?

Our response: We corrected "sing" with "single".

L150: A two-way analysis of variance (ANOVA) was

Our response: Corrected.

L153: delete "(SAS institute, 2013)" (this is already mentioned in the reference [33])

Our response: Corrected.

L153-154: replace the second "(SAS Institute, 2013)" with "[33]"

Our response: Corrected.

L176: "Population parameters were as follows:" ; please add the corresponding reference for these formulas

Our response: Corrected.

L193: of each treatment group showed

Our response: Corrected.

L197: in each treatment group of

Our response: Corrected.

L199: the quality of this Figure 1 should be improved

Our response:

Thank you for the comments. We changed this figure to a higher resolution.

L226, L231, L239, L244, and L261: Means followed

Our response: Corrected.

L267: in the DISCUSSION section, there is a clear lack of info supported with references (references are used only in the first paragraph !!). Please add more relevant references (and related info from other countries / other *B. tabaci* biotypes - comparisions with your findings) to other parts of this section that needs to be improved to reach an acceptable level for publication. In this context, please note that what you wrote "Life table parameters of *B. tabaci* have been previously reported regarding different putative species, host plants, or temperatures [12, 13, 34-38]." is a too general statement, so I'd suggest you to give separately (different sentences) more details linked to this general statement and to the supporting references used here (12, 13, 34-38])

Our response:

Thank you for the comments on our study. The references [12, 13, 34-38] have been revised with other papers [36-45] (see line 276 in the revision).

L295-296: "Insecticide resistance might also play a role in the prevalence of genetic cluster 2" ; please add references to support this

Our response:

We added this reference [50] (Line 309 in the revision).

The Figure page 30 is of bad quality, so it should be improved for more clarity to the reader

Our response:

Thank you for the comments on our study. We changed this figure to a higher resolution.

**Reviewer #2:**

The title doesn’t match with why the study was performed.

Our response:

Thank you for the comments on our study. We revised the title more adequately to represent the manuscript than before. Now, the revised title is “Comparison of life history parameters of two different genetic clusters of *Bemisia tabaci* MED (Hemiptera: Aleyrodidae) through single and cross mating”.

The provided abstract does not adequately represent the manuscript.

Our response:

Thank you for the comments on our study. We revised some parts of the manuscript more adequately to represent the manuscript than before.

The introduction is poorly written for example in lines 48-49 “Potentially different insecticide resistance of these genetic clusters, if any, might be partly involved in genetic cluster change » authors do not report the two important characteristics concern the harbouring of endosymbionts and the transmission of plant viruses that can modify whitefly feeding behaviour. Consider the two references below:

- Kirk, H., Dorn, S. & Mazzi, D. Molecular genetics and genomics generate new insights into invertebrate pest invasions. Evol. Appl. 6, 842–856 (2013).

- Liu, B. M. et al. Multiple forms of vector manipulation by a plant-infecting virus: *Bemisia tabaci* and tomato yellow leaf curl virus. J. Virol. 87, 4929–4937 (2013).

Our response:

Thank you for the comments on our study.

As suggested, we added a new statement and corresponding references to introduction as follows (Lines 51-52): Plant virus transmission rates and endosymbionts can also affect host’s biology and physiology, thus being able to change genetic cluster [7, 8] (Legg et al., 2002 Pan et al., 2012).

In fact, we conducted follow-up studies to determine whether the presence or absence of tomato yellow leaf curl virus (TYLCV), a representative virus mediated by *B. tabaci*, and *Wolbachia* was associated with the genetic cluster changes. However, neither TYLCV nor *Wolbachia* was associated with the changed genetic clusters. Of the total populations, only 2016 BY population was infected with *Wolbachia*. Although we did not report these results in original manuscript, we have added new statements explaining the results to the discussion section (Lines 301-307) along with supporting information (S2 and S3 Tables). The newly added statements are: “Beside the life history traits, plant virus transmission rates and endosymbionts are known to affect the biology and physiology of their host. Therefore, we investigated whether the presence of absence of tomato yellow leaf curl virus (TYLCV), a representative virus mediated by *B. tabaci*, and *Wolbachia* was associated with the genetic cluster changes. However, neither TYLCV nor *Wolbachia* was associated with the changed genetic clusters (S2 and S3 Tables).”

Also one of the follow-up research related to insecticide resistance mentioned in the introduction is currently under review after submitting to the journal of Pest Management Science (Manuscript number: PM-20-1332).

The Materials and Methods section.

Table 1 need major work :

-Columns 2 and 5 Treatment and crosses, male and female are represented by m and f and also by ♀ and ♂ symbols

Our response:

We changed symbols in columns 5 with "f and m" and added a footnote at the bottom of table 1 (Line 134).

- Column 3 culture type, what does it mean sing cluster?

Our response:

We changed "sing" with "single".

- Column 4 Mating method, the words single and single crossing are somewhat confusing. The single crossing should be replaced by cross mating.

Our response: Corrected.

Lastly, the results and discussion sections seem more like a thesis-style

The discussion needs major rewriting to improve clarity indeed authors gave only 4 references (line 271). As mentioned earlier, significant number of questions left unanswered such the role of endosymbiotic bacteria and virus transmission in the whitefly behavior.

Our response:

Thank you for the comments on our study. The references of line 276 have been revised with papers [36-45]. In addition, we revised the discussion section by providing detailed information on various factors potentially affecting genetic changes and new results. The newly added statements are: “These studies were conducted to determine the effect and correlation of various conditions. The comparison of these studies demonstrated that the correlation between life table parameters of *B. tabaci* and diverse environments are influenced high. (Lines 276-279)”

**Reviewer #3:**

**Main Comments:**

Stating that I am not a geneticist, I admit I do not know whether the terminology used in the paper is adequate. However, as a "non-geneticist entomologist", I have the feeling that the term "convergence" is not the most convenient to indicate the phenomenon of prevalence of Cluster C1 over Cluster C2. With reference to two distinct elements, A and B, the term convergence indicates, in my opinion, a reciprocal dynamism of the two elements, with each one going in the direction of the other; while, in the specific case presented in this paper, one element prevails over the other. In this case, can the term "convergence" be used? Or would it not be better to use the term "displacement" to indicate the dominance of Cluster C1 over Cluster C2 and the progressive reduction of the latter?

Please, be aware, this does not want to be a criticism but simply a doubt and “food for thought”.

Anyway, if my doubts on the term “convergence” are accepted by the Authors, they should modify accordingly the term in many lines of the paper, such as: lines 19, 32, 44, 274, 281, 299.

Our response:

Thank you for the main comments on our study. We agree with the reviewer on these important points. We revised the term to ‘displacement’ (Lines 19, 32, 46, 283, 289, and 313 in the revision).

**Specific Comments:**

1. Line 39: Add “,”after “Aleyrodidae)”.

Our response: Corrected.

2. Line 40: literature cited [1-3] is almost scarce and of limited importance for the phrase quoted. Above all, paper no. 1 (Chen et al., 2004) and no. 3 (Tsagkarakou et al., 2012) refer to extremely detailed and particular aspects (for both host plants and diseases caused, as well as for geographic distribution cited); therefore, they are not well suited to being cited as example of references to the high harmfulness of *B. tabaci* at a broad geographical level and on a wide range of crops (these two citations could be deleted, eventually). Paper no. 2 (Navas Castillo et al., 2011) is better suited to this aim and should be maintained, definitively. But many other papers could be added, dealing with harmfulness of *B. tabaci* worldwide, on wide geographical areas and cropping systems.

Our response:

Thank you for the specific comments on our study. References [1] and [3] have been changed to Oliveira et al., 2001, De Barro et al., 2011.

3. Line 271: literature cited [12, 13, 34-38] is not satisfactory and updated; in general, it is not giving the right and complete idea of the huge scientific production on biology of different putative species of *B. tabaci* on different host plants and at varying temperatures. I strongly suggest to include also the following references (or at least the most recent ones, citing most of the previous papers).

Our response:

Thank you for the specific comments on our study. The references of line 276 have been revised with the papers you had provided [36-45].
