# Supplementary material for: Comparison of life history parameters of two different genetic clusters of Bemisia tabaci MED (Hemiptera: Aleyrodidae) through single and cross mating
Source: PLoS One. 2021 Mar 26;16(3):e0248819. doi: 10.1371/journal.pone.0248819 (PMC7997046; doi:10.1371/journal.pone.0248819)
Supplement: Attachment — Submitted filename: Response letter.docx [file pone.0248819.s008.docx]

**Dear Editor and reviewer.**

We are grateful for the reviewer’s valuable comments to improve our manuscript. We improved our manuscript significantly by incorporating the reviewer’s comments and suggestions. We have colored revised parts in red in the manuscript. Followings are our point-by-point responses to the comments:

**Reviewer #2:**

I think the MS can be accepted for publication in PlosOne journal as authors provided a point-by-point response to my specific comments.

Thank you for the comments on our study.

**Reviewer #3:**

VERY MINOR CHANGES STILL REQUIRED:

Thank you for the specific comments on our study.

Line 19: please change “displacement” with “have been displaced”.

Our response: Corrected.

Line 46: please change “displacement” with “have been displaced”.

Our response: Corrected.

Line 283: please change “displacement of” with “displacement to”.

Our response: Corrected.

**Reviewer #4:**

This manuscript compares the life cycle parameters of two different genetic clusters (one dominant the other) of the *Bemisia tabaci* MED species; it is very clear and well written.

I have only a few comments concerning the genetic analysis:

- In your section of materials and methods for population genetic analysis using STRUCTURE software, I would like to know what model did you use regarding populations mixtures? (ie. “No admixture” or “admixture”). Please indicate this in your M and M section.

Our response:

Thank you for the specific comments on our study. We added a new sentence additional explanation (Lines 143 – 144) as follows: “We used an ancestry model allowing for admixture and correlated allele frequency among treatments.”

- You took only “females” to perform the genetic analysis and, as a result, I expected to see only “hybrid patterns” in your “C1C1 crossings” or at least some, especially with respect to the observed “purity” of your parental lines (at least from C1 on both host plants) in the Bayesian results. I was very surprised to see that each population seems to be so different from the other, looking like different “species” (ie. almost no gene flow could be seen between individuals) despite the fact that in your crossings you obtained hybrids from both populations (biology experiment). You need to comment that in your discussion section and explain why.

Our response:

Thank you for the comments on our study. As *B. tabaci* is a haplo-diploid species in which males are haploid and produced from unfertilized eggs, all microsatellite genotyping was done using, diploid females (Mckenzie et al., 2012; Tsagkarakou et al., 2012).

The contents relevant to your comments are mentioned in lines 30-32, 219-221, and 299-301 of the manuscript (about maternal and paternal inheritance), and this experimental data (microsatellite and life table analysis) alone does not provide any direct clue for the driving force for the rapid displacement. With this in mind, further follow-up studies are needed to find out more accurate causes. We edited “Biological and life history parameters” to “Biological, life history parameters, and clusters proportion” (Lines 299-300).

- Since you’re using population genetics tools, one would expect that you would also present in the sup data, at least, the basic genetic indeces: allelic richness (na), expected/observed heterozygosity (He/Ho), and the fixation index (Fis) for your tested populations. Indeed, it would be interesting to see the degree of homozygosity obtained in your pure parental populations (even in the small sample you took).

Our response:

Thank you for the comments on our study. We have added a new supporting information (S1 Tables). Also we added a new sentence additional explanation (Lines 201-202): “The genetic diversity indices obtained from all the eight different microsatellite loci of *B. tabaci* MED screened are given in S1 Table.” Therefore, the existing Supporting table number has been changed (S1 is changed S2, S2 is changed to S3, and S3 is changed to S4).
